# PCB-containing wood floor finish is a likely source of elevated PCBs in residents' blood, household air and dust: a case study of exposure

**DOI:** 10.1186/1476-069X-7-2

**Published:** 2008-01-17

**Authors:** Ruthann A Rudel, Liesel M Seryak, Julia G Brody

**Affiliations:** 1 Silent Spring Institute, 29 Crafts Street, Newton, MA 02458, USA; 2 Division of Environmental Health Sciences, College of Public Health, The Ohio State University, 320 West 10th Ave., Columbus, OH 43210, USA

## Abstract

**Background:**

Polychlorinated biphenyls (PCBs) are persistent pollutants identified worldwide as human blood and breast milk contaminants. Because they bioaccumulate, consumption of meat, fish, and dairy products predicts human blood concentrations. PCBs were also used widely in building materials, including caulks and paints, but few studies have evaluated the contribution of these exposures to body burden.

**Methods:**

In an earlier study, we detected PCBs in indoor air in 31% of 120 homes on Cape Cod, MA. Two of the homes had much higher concentrations than the rest, so we retested to verify the initial finding, evaluate blood PCB concentrations of residents, and identify the PCB source.

**Results:**

Air and dust concentrations remained elevated over 5 years between initial and follow-up sampling. Blood serum concentrations of PCBs in residents of the homes were generally elevated above the 95^th ^percentile of a representative sample of the US population. Serum concentrations in residents and air and dust concentrations were especially high in a home where a resident reported use of PCB-containing floor finish in the past, and where the floor of one room was sanded and refinished just prior to sample collection.

**Conclusion:**

This case-study suggests that PCB residues in homes may be more significant contributors to overall exposure than diet for some people, and that use of a commercially-available PCB-containing wood floor finish in residences during the 1950s and 1960s is an overlooked but potentially important source of current PCB exposure in the general population.

## Background

Polychlorinated biphenyls (PCBs) are persistent organic pollutants and have been identified worldwide as human blood and breast milk contaminants. They are established developmental neurotoxicants in humans [1,2], with elevated prenatal PCB concentrations being associated with cognitive deficits in children [1,3-5]. PCBs are also associated with thyroid toxicity, effects on immune, reproductive, nervous, and endocrine systems, and cancer effects including breast cancer [6-10].

PCBs were primarily used in electrical equipment and fluorescent lighting fixtures prior to 1977, but can also be found in other products manufactured before 1977 such as plasticizers, surface coatings, inks, adhesives, flame retardants, pesticide extenders, carbonless duplicating paper, paints, wire insulators, caulking materials, elastic sealants, and heat insulation [6]. Because of the tendency of PCBs to bioaccumulate, several studies have concluded that consumption of meat and dairy products, as well as processed foods and fish, contributes significantly to overall intake of PCBs [11-15].

It is likely that major contributors to PCB exposure vary across the population depending on proximity to specific sources. For example, studies of Great Lakes fish eaters have shown that fish consumption is a significant predictor of blood PCB concentrations [16,17]. Use of PCB-contaminated caulk in commercial buildings and schools, which appears to be common in pre-1980 construction [18,19], has been shown to increase blood PCB concentrations, especially for lower-chlorinated congeners [20,21]. A few studies have proposed that multiple pathways contribute significantly to total human PCB exposure. Currado and Harrad determined that inhalation could account for 6 to 64% (mean 25%) of overall human PCB exposure [22].

In 2003 we reported results from a household exposure study of 120 homes on Cape Cod [23]. We tested for 89 target chemicals, mostly endocrine disrupting compounds (EDCs), and detected 52 chemicals in air and 66 in dust. Three PCB congeners were among the target chemicals: PCB 52, PCB 105, and PCB 153. We detected PCBs in air (>1 ng/m^3^) from 38 homes (32%) and dust (>0.2 μg/g) from 22 homes (18%) [23]. PCBs were detected in air in 76% of homes where it was detected in dust. Two homes had much higher air and dust PCB concentrations (sum of PCB 52, 105, 153 in air 23 and 35 ng/m^3^; dust 21 and 68 μg/g) than the rest of the study sample (max of sum PCB 52, 105, 153 in air 14 ng/m^3^; median < 3 ng/m^3^; max in dust 8 μg/g, median < 0.6 μg/g). These two homes were retested in an attempt to verify the initial finding, evaluate blood PCB concentrations of residents, and identify the source of PCBs with an ultimate goal of recommending potential exposure reduction techniques to the homeowners.

## Methods

Human research review committees at Brown University, Providence, RI, and at the Shattuck Hospital, Boston, MA, approved study protocols and all participants provided informed consent prior to sampling.

The 120 participants of the Cape Cod household exposure study were female breast cancer cases or age-matched controls who had participated in the case-control portion of the Cape Cod Breast Cancer and Environment Study [24] and had lived in their home at least 10 years at the time of original sampling (1999–2001). The average age of the women in the exposure study was 75 (median 77). In 2004–2005, new air and dust samples were collected from the 2 homes with high PCBs, and a visual survey of each house and the general area around the 2 homes was conducted to identify any potential sources of PCBs (e.g., electrical or industrial equipment, fluorescent lights, industrial window sealant, etc.). Residents were also asked about electrical and mechanical hobbies, businesses, historical land use, flooring, lighting, and older electrical equipment.

Sampling took place in single-family owner-occupied homes on Cape Cod, Massachusetts. Cape Cod is a coastal peninsula consisting of 15 towns in Southeastern Massachusetts. The area has very little industry and is considered a summer vacation destination. Follow-up air and dust samples were collected in winter 2004–2005, 4–6 years after the initial samples were taken from these homes, and analyzed using the same methods reported previously [23,25]. Briefly, air samples were collected over 24 hours by drawing air at 8–9 L/min through a personal pesticide sampling cartridge (University Research Glassware, URG). URG cartridges were fitted with a quartz fiber filter followed by XAD-2 resin sandwiched between two polyurethane foam (PUF) plugs. The total volume of air sampled ranged from 10–14 cubic meters.

Dust samples were collected using a Eureka Mighty-Mite vacuum cleaner, modified to collect dust through a Teflon crevice tool into a cellulose extraction thimble (Whatman Inc., Clifton, NJ). Dust sample collection did not begin until the air sample collection was complete. Sample collection was accomplished by slowly and lightly drawing the crevice tool just above the surface of rugs, upholstery, wood floors, windowsills, ceiling fans and furniture in each room. Sampling was conducted in the most frequently-used rooms of the house, usually 4–5 rooms and including hallways. Cellulose thimbles containing dust were placed in cleaned glass sample jars with Teflon lined lids (Environmental Sampling Supply, Oakland, CA). URGs and dust samples were stored at -4°C until they were shipped overnight on dry ice to Southwest Research Institute (SWRI) for analysis. Prior to extraction, dust was tapped out of the thimbles, weighed, and sieved to < 150 microns.

Chemical analysis of air and dust samples was conducted at SWRI, San Antonio, TX as described in [23,25]. Each XAD-2/PUF/filter was soxhlet extracted in 6% ether in hexane. Each sieved (< 150 um) dust sample was spiked with surrogate, equilibrated, then soxhlet extracted using 6% ether in hexane. The extracts were concentrated and cleaned by running through a florisil column. Analysis was performed using an Agilent 6890/5973 GC/MS in selected ion monitoring (SIM) mode. The GC/MS instrument was scanned to monitor the following ions: PCB 52 (290,294), PCB 105 (324,328), PCB 153 (362,358). The base peak ion was used as the quantification ion for each compound. Quantification was performed using d12-labeled PAHs as internal standards. PCBs were not detected in air matrix blanks or dust extraction blanks. Congener sums assumed a value of 0 for values below the detection limit.

Within one month of air and dust re-sampling, blood samples were collected from residents of the two homes with elevated PCBs to better describe personal exposure. Serum samples were analyzed for 33 PCB congeners along with several other organochlorines (e.g., p,p'-dichlorodiphenyl dichloroethylene, or 4,4'-DDE) at the Environmental Chemistry Laboratory, Division of Analytical Chemistry, Massachusetts Department of Public Health State Laboratory Institute in Jamaica Plain, MA. The analysis followed the AOAC standard method for polychlorinated biphenyls in serum [26] and involved extraction with hexane/ethyl ether, silica column cleanup with hexane elution, and analysis by dual column gas chromatography with electron capture detection as described in Brock et al. [27]. Standard operating procedures for all analyses are available from the authors. Results were reported as ng/g lipid and detection limits were about 15 ng/g lipid. Any detects in a reagent blank prepared and analyzed with the samples were subtracted from sample results. Congener sums assumed a value of 0 for below detection limit values. These sums underestimate total PCB concentrations in serum because 1) zero very likely underestimates the concentrations of some congeners that may be present below detection limits, and 2) only 33 of 209 PCB congeners were analyzed.

## Results

### PCBs in Indoor Air

PCBs in indoor air remained elevated on re-testing in both homes with high PCB concentrations in the initial study (Table 1). Measurements were taken in the living area of the homes initially, and in the living area and bedroom at retest. In home #1, living area concentrations remained high. PCBs were not detected in the bedroom, which was on the second floor of the home, but the limit of detection was elevated because of the small sample volume (see Table 1). Home #2 was small and the living area and bedroom were close together – concentrations were similar in both rooms. Both retest samples were collected during the winter, while initial samples from home 1 were collected in winter and home 2 in summer.

**Table 1:** PCB concentrations in indoor air, house dust, and residents' serum in two Cape Cod homes

		Indoor air(ng/m^3^)^a^		House dust (micrograms/gram)^a^		Serum (ng/g lipid)		
Home	Participant	Initial PCBs, LR [52,105,153]	Follow-up PCBs LR (BR) [52,105,153]	Initial PCBs [52, 105, 153]	Follow-up PCBs [52, 105, 153]	Total PCBs (# congeners detected)^b^	Sum 10 PCB congeners^c^	4,4'-DDE^d^

1	1-A	23 [19, 4, <2]	21 (<68) [10, 2.9, 7.7]	21 [1.1, 14, 5.8]	190 [15, 53, 119]	1630 (13)	1520	848
	1-B^e^					993 (11)	967	208
2	2-A	35 [25, 6.7, 3.6]	8.0 (13) [8, <2.8, <2.8]	68 [16, 35, 17]	140 [37, 34, 69]	760 (16)	652	292
	2-B					179 (5)	179	96

Abbreviations: PCB, polychlorinated biphenyl; LR, living room; BR, bedroom; ND, not detected; DL, detection limit

^a^PCB concentrations reported as sum of PCB 52, 105, 153 in air or dust (<DL values set to 0), followed by results for the 3 individual congeners (PCB 52, 105, 153)

^b^Sum of the concentrations of all PCB congeners detected in serum (of 33 tested). Values < DL set to 0. Individual congeners reported in Additional File 1

^c^Limited to the 10 PCB congeners also measured in NHANES. Values < DL set to 0

^d^Included as an alternate bioaccumulative, persistent organochlorine

Concentrations of PCBs in air were high compared with another Massachusetts study and well above EPA health-based guidelines (Table 2). In our study, the sum of PCBs 52, 105, and 153 ranged from 8 to 35 ng/m^3 ^in the living areas of the two homes. Vorhees et al. found a maximum of 7.3 ng/m^3 ^for the sum of those 3 congeners in 15 homes intended to represent "background" PCB concentrations in Southeastern Massachusetts, close to Cape Cod [28]. Congeners 52, 105, and 153 represent 9% of the sum of all 65 congeners measured in air in the Vorhees study, and this proportion is stable across many homes (standard deviation of 3% for 70 measurements in 34 homes) (unpublished observation). Therefore, it is likely that total PCB concentrations in the two Cape Cod homes are considerably higher (>10X) than the sum of the 3 measured congeners. The EPA health-based screening value of 3.4 ng/m^3 ^for total PCBs is intended to be protective for health effects following long-term exposure [29]. The sum of 3 PCB congeners in indoor air in the Cape Cod samples exceeds this health-based screening value for ambient air by about a factor of 10, and total PCBs are likely substantially above the guideline.

**Table 2:** Comparison values and context for Cape Cod PCB levels.

		Indoor air (ng/m^3^)	House dust (μg/g)	Serum (ng/g lipid)
EPA health-based guideline^a^		3.4	0.22	
Maximum for 16 MA homes^b (PCB 52+105+153 only)^		7.3	0.6	
Maximum for 16 MA homes^b (sum of 65 PCB congeners)^		51	3.6	
Maximum for 1046 U.S. homes^c (sum of PCB 105+153)^			10	
NHANES PCBs^d^	median			267
	75th percentile			394
	95th percentile			715
	maximum			1466
NHANES 4,4'-DDE^e^	median			692
	75th percentile			1314
	95th percentile			2569
	maximum			6510

abbreviations: PCBs, polychlorinated biphenyls; NHANES, National Health and Nutrition Examination Survey

^a^EPA Health-based guidance value for total PCBs – EPA Region 9 Preliminary Remediation Goals for ambient air and residential soil (2004).

^b^Maximum concentration of the sum of PCB congeners tested in a study of 16 homes (15 homes for dust) intended to represent background PCB levels in Southeastern, MA (Vorhees et al. 1997 and 1999). On average PCBs 52+105+153 represent 9% of the 65 congeners in air samples from this study and 12% of the congeners in dust.

^c^Sum of the maximum concentrations of PCBs 105 and 153 in a non-hodgkin lymphoma case-control study of 1046 homes in the U.S. (Colt 2005).

^d^NHANES 1999–2002 data for white women over age 59 (n = 319) – sum of 10 PCBs measured on Cape Cod; non-detects set to 0. The 95th percentile for white men over age 59 (n = 295) is 679 ng/g lipid.

^e^NHANES 1999–2002 data for white women over age 59 (n = 319) – 4,4'-DDE included as an alternate bioaccumulative, persistent organochlorine

### PCBs in Dust

PCBs in house dust remained elevated or increased on re-testing in both homes with high PCB concentrations in the initial study (Table 1). Concentrations of PCBs in house dust samples were high compared with the Vorhees study in Massachusetts [30] and a large study with over 1000 samples collected across the US [31]; and were well above EPA health-based guidelines (Table 2) [29]. In our study, the sum of PCBs 52, 105, and 153 ranged from 21 to 190 μg/g dust in the two homes. In house dust samples in a set of 15 homes intended to represent background PCB concentrations in Southeastern Massachusetts, Vorhees et al. found a maximum concentration of 0.6 μg/g for the sum of PCB 52, 105, and 153, and these 3 congeners made up 12% of the sum of 65 congeners reported ([30]; unpublished observation). In 1000 vacuum cleaner bag samples collected from homes in Iowa, Seattle, Detroit, and Los Angeles as part of a case-control study of organochlorines and non-Hodgkin lymphoma, Colt et al. reported a maximum concentration of the sum of PCBs 105 and 153 in dust of 10 μg/g [31] compared with our 190 μg/g. Although no health-based screening values have been developed for PCBs in house dust, the EPA screening values for residential soil may be a reasonable comparison, since exposure assumptions for house dust and residential soil are generally similar in EPA risk assessments. The EPA Region 9 soil screening value may be slightly higher (less protective) than a screening value for dust exposure would be, because infants are assumed not to be exposed to soil, while they are expected to have high exposures to house dust. PCB concentrations in house dust in this study exceed health-based screening values for residential soil developed by EPA by a factor of 860 without adjustment for the expected total PCB represented by the three congeners we measured. The EPA health-based screening value of 0.22 μg/g for total PCBs is intended to be protective for health effects following long-term exposure [29].

### PCBs in Blood Serum

The concentrations of PCBs in the participants' blood serum were elevated compared with those reported in the US Centers for Disease Control and Prevention's *3*^*rd *^*National Report on Human Exposure to Environmental Chemicals *[32] – a survey of exposure in US residents conducted as part of the National Health and Nutrition Examination Survey (NHANES) (Tables 1 and 2). We measured the PCB concentrations in two residents from each home, a total of three women (participants 1A, 2A, and 2B) and one man (1B). It has been established that age, sex, and race are predictors of human PCB concentrations [15,33]. Therefore, we also compared our blood data with a subset of the NHANES 1999–2002 reference data similar to our demographic group of white women (or men) over the age of 59 (Table 2). Comparing results for the sum of 10 PCB congeners measured in both NHANES data and the Cape Cod samples shows that 3 of the 4 Cape Cod residents are above the 95^th ^percentile of the NHANES study sample. In fact participant #1A has total PCBs above the maximum reported in NHANES 1999–2002 for the set of 319 white women over the age of 59 (Table 2). Participant 2B, who has blood PCB concentrations below the NHANES median, had been living in the home less than 6 months at the time of the sampling (other participants had lived in homes > 10 years). Individual congener results for serum samples are included in Additional File 1.

4,4'-DDE measured in these blood samples serves as an indicator of body burden of persistent, bioaccumulative organochlorine contaminants which, like PCBs, tend to be higher in older persons and those with high intake of animal fats. DDE concentrations were not elevated in these four participants – three tested below the 50^th ^percentile of the older white women (or men) subset of NHANES, and participant #1A was between the 50^th ^and 75^th ^percentiles (Tables 1 and 2).

### Interviews about likely PCB sources

In the Cape Cod Breast Cancer and Environment Case-Control Study, participants were asked about their past and present fish-eating habits. No data are available for participant #1A. Participant #2A, who had elevated PCBs in her home and her blood, reported eating saltwater fish from Cape Cod waters less than once per month but more than 6 times per year. She reported not eating Cape Cod freshwater fish, seafood, or lobster on a regular basis (more than 6 times per year).

**Figure 1:**
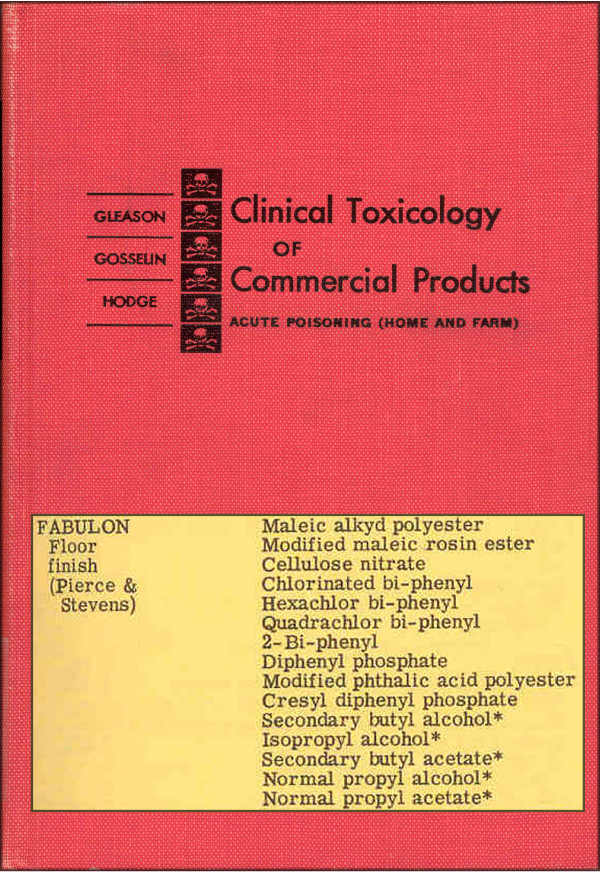
Entry for Fabulon floor finish from 1957 edition of Gleason et al. *Clinical Toxicology of Commercial Products *lists PCBs among the ingredients

During participant interviews and household surveys to identify potential sources of PCBs, one resident in home #1 recalled having used a floor finish called Fabulon on hardwood floors throughout the 1950s and 1960s. We consulted an out-of-print set of reference books designed for poison control centers and medical professionals, *Clinical Toxicology of Commercial Products*, which reported that in 1957 Fabulon's formula contained chlorinated biphenyl, hexachlor bi-phenyl, and quadraclor bi-phenyl, as shown in Figure 1[34]. By 1969, PCBs were no longer listed as part of the Fabulon formula, according to a later edition of the same resource book [35]. The resident of home #2 did not recall what particular floor finish was used in the 1950s and 1960s, but the home had wood floors that had not been refinished in many years. No other likely sources of PCBs were identified in our home surveys and questionnaires.

The resident in house #1 also informed us that some of the floors in the house had been sanded and refinished since the 1960s, while the Fabulon finish remained on other floors in the home. Interestingly, the floor in one of the rooms in home #1, the home with the very high dust concentrations on retest and the resident with the serum PCBs above the maximum reported in NHANES 1999–2002, had been sanded and refinished immediately prior to the 2005 blood sampling and air and dust re-sampling. Because sanding of wood floors liberates a large amount of dust, the sanding of a Fabulon-coated wood floor may account for the unusually high concentrations we measured in dust (more than 8 times higher than first dust sample) and blood observed in the female resident of home #1. Blood PCB concentrations in a male resident of home #1 were also quite elevated (above the 95^th ^percentile) relative to the comparison group of older white men in NHANES.

## Discussion

In this study we have identified 2 Cape Cod homes with air and dust PCB concentrations much higher than in 118 other homes in the same region of the US and much higher than reported in homes in other parts of the US [23,28,30,31]. Elevated air and dust concentrations persisted over 5 years between initial and follow-up sampling. Follow-up samples of blood serum showed that concentrations of PCBs in residents of these homes were also generally elevated above the 95th percentile of NHANES, except in one resident who had moved into the home during the 6 months prior to sampling. Serum and dust concentrations were especially high in residents of home 1, where a PCB-containing floor finish was reportedly used in the past, and where the floor of one room was sanded and refinished within a week prior to the sample collection. No other likely sources of PCBs were identified in either home by inspection, and wood floors in home 2 had not been refinished in many years. Based on these observations, we hypothesize that the source of PCBs in these two homes, and in the residents of those homes, is PCB-containing wood floor finish such as the Fabulon product reported to have been used in home #1. In addition to the listing of Fabulon in Gleason et al. [34] as containing chlorinated biphenyls, other sources indicate that PCBs were used in varnishes and paints in the 1950s and 1960s [36,37]. We have not confirmed that the wood floors are a source of PCBs in these homes. Such a confirmation could create regulatory and disclosure obligations that pose significant challenges to individual home owners. We are working to develop helpful and appropriate follow up in this context.

Earlier studies of PCBs in humans have often focused on diet as the primary source of PCB exposure. Several of these studies found that people who ate greater amounts of Great Lakes fish – known to contain large amounts of PCBs – had higher PCB serum loads than people who ate minimal amounts of contaminated fish [12,16,17]. However, these studies never examined exposure to PCBs from alternate sources like household products contributing PCBs to the air and dust.

It is unlikely that the body burdens of PCBs we observed would be coming from the participants' diets. First, PCBs were found at very high concentrations in the blood of participants who had high concentrations in their air and dust. Second, these participants do not have similarly high concentrations of other fat-soluble persistent organochlorines in their blood, although the same constituents of diet that contribute PCBs are presumed to be the major source of exposure to banned organochlorine pesticides. Participant #1A's blood concentration of 4,4'-DDE is between the 50^th ^and 75^th ^percentile for older white women in NHANES. All other participants are well below the 50^th ^percentile for the older white women (or men) subset of NHANES (Tables 1 and 2).

Analysis of the specific congeners in the serum (given in Additional File 1) also indicates that the 3 participants who have been long time residents in these homes have congener profiles that are more similar to workers exposed to commercial mixtures of PCBs while the participant who recently moved into the home has a serum congener profile more consistent with general population exposure through dietary sources. Due to the transformation of PCBs as they move through the environment, a different distribution of congeners is observed in individuals exposed to PCBs through fish consumption compared with individuals exposed in an occupational setting. Freels et al. [38] compared congener profiles in capacitor plant workers occupationally exposed to PCBs and Great Lakes sport-fish consumers, with about 200 individuals in each group. They found that the ratio of congeners (74+153+201)/(74+153+201+138+180) correctly classified more than 99% of the individuals in the study, with fish-eaters having a ratio in the range 0.26–0.49 (mean 0.39) and capacitor workers having a ratio in the range 0.42–0.95 (mean 0.68). For our participants, the ratios were 0.54, 0.50, 0.47, and 0.43; so 2 of our participants were outside the range observed in the fish eaters and 2 were in the area of overlap between the two groups, with the lowest ratio being observed in the participant who moved to the home recently.

Wingfors et al. [39], compared serum congener profiles between Swedish workers removing PCB-containing caulk and the general population in Sweden. The ratio of certain congeners to congener 153 was shown to differentiate occupationally exposed individuals from the general population whose exposure is primarily through diet. In our set of 4 participants, the three longer-term residents had ratios more like the occupationally exposed individuals compared to the one who moved in more recently. Our exposed participants' serum was particularly enriched with congeners 74, 99, 118, and 105 (relative to 153); and diminished in congener 180 (relative to 153). However, the actual ratios were quite different between our participants and the Swedish cohort, suggesting the exposure source is a different PCB mixture. A limitation of our study is that only 3 PCB congeners were analyzed in the air and dust samples, limiting our ability to compare congener profiles in the environmental media with serum.

## Conclusion

Although background concentrations of PCBs in human blood may be closely correlated with diet, the case-study data presented here suggest that some people may be exposed to PCB residues in their homes that are more significant contributors to their overall exposure than diet. The use of a commercially-available PCB-containing wood floor finish in residences in the 1950s and 1960s is an overlooked but potentially important source of PCB exposure in the general population. In addition, potential exposure to floor sanders may be significant and studies to evaluate blood PCB concentrations among this group are needed. Taken together with the prevalence of PCB-containing caulk in commercial buildings and schools [18,19], and the observation by Jamshidi et al. that PCBs in outdoor air originate from indoor sources [40], these findings suggest that the exposure potential posed by historic use of PCBs in building materials may be significantly underestimated.

## Abbreviations

p,p'-dichlorodiphenyl dichloroethylene, 4,4'-DDE; endocrine disrupting compounds, EDCs; Environmental Protection Agency, EPA; Massachusetts, MA; National Health and Nutrition Examination Survey, NHANES; not detected, ND; polychlorinated biphenyl, PCB; polyurethane foam, PUF; United States, US; University Research Glassware, URG; Southwest Research Institute, SWRI

## Competing interests

The author(s) declare that they have no competing interests.

## Authors' contributions

RR and JB conceived the idea for the follow-up study and directed it. RR carried out some follow-up sampling with technical support, interviewed the participants about potential sources of PCBs in their homes, and made the discovery that PCBs had been a major component of the wood finish described by the study participant. LS drafted the manuscript, and RR revised it. All three authors contributed to deliberations about ethical and policy implications of this finding.

## Supplementary Material

Additional file 1 Concentrations of PCB congeners measured in residents' serum. The data provided represent individual PCB congener measurements for each of the four blood samples discussed in the paper. Click here for file
